# A New Accessible Six-Strand Flexor Tendon Repair Technique: A Four-Group Comparative Study on a Porcine Model

**DOI:** 10.3390/jcm15197449

**Published:** 2026-09-24

**Authors:** Szymon Manasterski, Michał Chęciński, Piotr Węgrzyn, Maja Smorąg, Marta Jagosz, Jędrzej Króliński, Patryk Ostrowski, Michał Bonczar, Ahmed Elsaftawy

**Affiliations:** 1 Plastic Surgery and Hand Surgery Department, St. Jadwiga Śląska Hospital, 55-100 Trzebnica, Poland; smanasterski@gmail.com (S.M.);; 2 Department of Anatomy, Jagiellonian University Medical College, 31-202 Kraków, Poland; 3 Youthoria, Youth Research Organization, 31-202 Kraków, Poland

**Keywords:** flexor tendon repair, six-strand suture, Elsaftawy technique, tendon biomechanics, porcine model

## Abstract

**Background:** Six-strand flexor tendon repairs offer superior biomechanical strength, yet commonly used techniques often require looped sutures or proprietary materials that limit global accessibility. The Elsaftawy repair technique was developed as a six-strand construct using universally available braided polyester, aiming to provide high strength without reliance on specialty sutures. **Methods:** Sixty porcine digital flexor tendons were randomized into four groups: Elsaftawy 6-strand, Jin Bo Tang 6-strand, FiberLoop^®^, and modified Kessler 4-strand. Core repairs only were performed by a single experienced surgeon. Tendons were biomechanically tested for 2 mm gap formation force, ultimate tensile strength, suturing time, and failure mechanism. **Results:** All 60 tendons completed biomechanical testing without specimen loss. The Elsaftawy and Jin Bo Tang repairs demonstrated comparable biomechanical performance and significantly greater resistance to 2 mm gap formation and ultimate tensile strength than the Kessler repair (*p* < 0.05). FiberLoop^®^ showed significantly lower gap resistance and ultimate load compared with both six-strand techniques but did not differ significantly from the Kessler repair. FiberLoop^®^ was the fastest technique to perform (*p* < 0.05), whereas Elsaftawy and Jin Bo Tang repairs failed predominantly by suture rupture, in contrast to the pullout-dominant failure pattern observed with FiberLoop^®^. **Conclusions:** The Elsaftawy repair uses an accessible suture and showed no statistically significant differences in the measured biomechanical outcomes compared with the Jin Bo Tang technique. Its initial mechanical performance and use of standard non-looped sutures support further investigation, including assessment of reproducibility across surgeons and clinical outcomes.

## 1. Introduction

Flexor tendon repair remains one of the most technically demanding procedures in hand surgery, requiring a balance between mechanical strength, minimal bulk, and compatibility with early rehabilitation protocols [1,2]. Early gap formation at the repair site has been consistently associated with increased gliding resistance, adhesion formation, and postoperative dysfunction [3]. Biomechanical evidence suggests that six-strand core suture techniques provide superior resistance to gap formation and rupture compared to traditional two- and four-strand repairs [4]. These multi-strand constructs, exemplified by the Jin Bo Tang looped technique [5], have facilitated the safe implementation of early active motion protocols and improved clinical outcomes.

Despite their mechanical advantages, six-strand repairs that rely on looped sutures, such as Supramid^®^ or FiberLoop^®^, face barriers to widespread adoption. Loop-configured sutures are not universally available, particularly in resource-limited settings, and are often cost-prohibitive. Additionally, some techniques, such as the Arthrex FiberLoop^®^ repair, require complex multi-step sequences and specialized training, increasing the learning curve and limiting feasibility in general surgical practice. Moreover, biomechanical studies have shown that while UHMWPE looped sutures offer high tensile strength, they are prone to failure by suture pullout if the core purchase or repair architecture is suboptimal [6].

Four-strand Kessler-type repairs remain popular due to their simplicity but demonstrate lower resistance to early gap formation and lower ultimate strength. Published values for 4-strand repairs vary widely depending on whether epitendinous augmentation is included, with gap forces typically ranging from 20–60 N [7,8,9].

The Elsaftawy repair was developed to address these limitations (Figure 1 and Figure 2). This six-strand technique employs a symmetric, multi-vector suture path with two pretensioning knots using standard, non-looped braided polyester suture, materials universally available in most operating rooms. By eliminating the dependence on specialty sutures and minimizing technical complexity, the Elsaftawy repair aims to deliver the mechanical advantages of six-strand constructs in a form that is reproducible, cost-effective, and widely accessible. This study compares the biomechanical performance of the Elsaftawy six-strand repair in 4-0 braided polyester configurations with three commonly used alternatives: the Jin Bo Tang six-strand looped repair, the four-strand modified Kessler repair, and the FiberLoop^®^ technique. The primary outcomes include resistance to 2 mm gap formation, ultimate tensile load, suturing time, and failure mechanism, using a standardized porcine flexor tendon model. By evaluating both performance and practicality, this investigation aims to determine whether the Elsaftawy technique can serve as a viable global alternative for high-strength, reproducible flexor tendon repair, particularly in settings where cost, material access, or technical training are limiting factors.

## 2. Materials and Methods

### 2.1. Specimen Preparation

Porcine digital flexor tendons were selected as the experimental model because their viscoelastic and structural properties closely resemble those of human flexor tendons [10]. Ethical approval was not required because the study was performed exclusively on ex vivo porcine flexor tendons obtained after routine slaughter. No live animals were used, and no animals were sacrificed specifically for this study. The ARRIVE 2.0 checklist was used to guide reporting, where applicable to the ex vivo study design (Table S1). Sixty superficial digital flexor tendons were harvested from skeletally mature domestic pigs (Sus scrofa domestica) within 12 h post-mortem at a licensed abattoir. Tissue was stored at 4 °C in saline-moistened gauze and dissected within 24 h. To preserve fiber integrity and avoid collagen crosslink disruption, specimens were never frozen. Each tendon was exposed under magnification, meticulously cleared of surrounding fascia and muscle, and standardized to a length of approximately 120 mm. Cross-sectional diameter was measured using a digital caliper; tendons exhibiting visible degeneration, fraying, or structural inconsistency were excluded.

### 2.2. Study Design

All tendons were sharply transected at their midpoint with a No. 11 blade to create a standardized complete laceration mimicking a Zone II injury. Samples were randomized using a computer-generated sequence into four repair groups, each representing a clinically relevant technique: (1) Elsaftawy 6-strand (4-0 coated braided polyester, PremiCron^®^, B. Braun, Melsungen, Germany)—six-strand configuration using standard non-looped braided polyester suture; (2) Jin Bo Tang 6-strand (4-0 polyamide, Supramid^®^, Resorba Medical GmbH, Nuremberg, Germany)—canonical six-strand looped repair using 4-0 Resorba Supramid^®^ as described by Tang et al. [5]; (3) Kessler 4-strand (4-0 coated braided polyester, PremiCron^®^, B. Braun, Germany)—modified four-strand Kessler repair constructed with 4-0 braided polyester; (4) FiberLoop^®^ UHMWPE (4-0 looped, FiberLoop^®^, Arthrex, Naples, FL, USA)—Arthrex FiberLoop^®^ repair performed using the manufacturer’s official 16-step technique.

### 2.3. Suturing Technique

All repairs were performed by a single fellowship-trained hand surgeon with Tang Level III expertise [11] to eliminate inter-operator variability. Repairs were conducted under ×3.5 loupe magnification. The following parameters were standardized: (a) Needle insertion angle: 45° ± 5°; (b) Purchase distance: 10 mm from each tendon edge; (c) Stitch depth: approximately 2 mm; (d) Core suture only: no epitendinous augmentation was applied to isolate core performance. Suturing time was measured with a digital chronometer beginning at the first needle insertion and ending after final knot trimming. Throughout repair, tendons were intermittently irrigated with isotonic saline to prevent desiccation (Figure 3).

### 2.4. Biomechanical Testing

Biomechanical evaluation followed the principles of ASTM D3039 [12] for soft biological tissues. Repaired tendons were mounted in a custom uniaxial tensile testing apparatus incorporating paired stainless-steel clamps, serrated jaws lined with emery paper to prevent slippage, a calibrated digital dynamometer (Sauter FK250/0.1 N, Germany), and a motor-driven linear actuator producing constant elongation. A 60 mm gauge length was maintained between clamps. Before tensile testing, tendons were pre-tensioned to 1 N for 30 s to eliminate slack. Tendons were distracted at a constant rate of 20 mm/min, with load–displacement data recorded at 10 Hz. The primary biomechanical outcomes included: (1) 2 mm gap formation force (N)—the load required to produce a 2 mm separation at the repair site, verified by high-resolution digital video and calibrated digital calipers; (2) Ultimate tensile strength (N)—the maximum load reached immediately prior to failure; (3) Failure mechanism, categorized as suture rupture or suture pullout (Figure 4).

### 2.5. Statistical Analysis

All data were imported into IBM SPSS Statistics v.29 for analysis. Descriptive statistics were expressed as mean ± standard deviation (SD). Normality was assessed using the Shapiro–Wilk test and Q-Q plots, whereas homogeneity of variance was evaluated using Levene’s test. Between-group comparisons were performed using one-way analysis of variance (ANOVA). Pairwise group comparisons were conducted using the Tukey HSD (Tukey–Kramer) test (α = 0.05), which adjusts for unequal group sizes. *p*-values < 0.05 were considered statistically significant.

## 3. Results

All 60 tendons completed biomechanical testing without specimen loss. All datasets demonstrated normal distribution (Shapiro–Wilk *p* > 0.05) and homogeneity of variances (Levene *p* > 0.05), validating the use of parametric testing. The established means for each subgroup are presented in Table 1.

### 3.1. Two mm Gap Formation Force

The Elsaftawy repair and the Jin Bo Tang technique did not differ significantly from each other (*p* = 0.992). Both techniques demonstrated significantly greater resistance to 2 mm gap formation compared with the Kessler repair (*p* = 0.0397 and *p* = 0.0191, respectively). FiberLoop^®^ 4-0 exhibited significantly lower resistance than both Elsaftawy and Jin Bo Tang repairs (*p* = 0.0009 and *p* = 0.0004, respectively) and did not differ significantly from the Kessler repair (*p* = 0.5711). Detailed results are presented in Table 2 and Figure 5.

### 3.2. Ultimate Tensile Strength

The Elsaftawy repair demonstrated the highest ultimate load, significantly exceeding both FiberLoop^®^ and Kessler repairs (*p* = 0.0011 and *p* < 0.0001, respectively). The Jin Bo Tang technique also showed significantly greater ultimate load compared with FiberLoop^®^ and Kessler repairs (*p* = 0.0009 and *p* < 0.0001, respectively). There was no significant difference between the Elsaftawy and Jin Bo Tang techniques (*p* = 0.9998). FiberLoop^®^ and Kessler repairs did not differ significantly from each other (*p* = 0.2517). Detailed results are presented in Table 3 and Figure 6.

### 3.3. Suturing Time

FiberLoop^®^ was the fastest technique, requiring significantly less suturing time than the Elsaftawy, Jin Bo Tang, and Kessler repairs (*p* = 0.0115, *p* < 0.0001, and *p* = 0.0316, respectively). No significant differences in suturing time were observed between the Elsaftawy, Jin Bo Tang, and Kessler techniques (*p* > 0.05 for all pairwise comparisons). Detailed results are presented in Table 4 and Figure 7.

### 3.4. Failure Mechanisms

Failure patterns differed between techniques. FiberLoop^®^ demonstrated predominantly suture pullout (73.3%), whereas rupture occurred in only 26.7% of cases. In contrast, both Elsaftawy and Jin Bo Tang techniques failed primarily through suture rupture (73.3%), with pullout observed in 26.7% of specimens. The Kessler repair showed exclusively rupture failure (100%). Detailed results are presented in Table 5.

## 4. Discussion

This study compared four clinically relevant flexor tendon repair techniques using a standardized porcine model to assess their resistance to early gapping, ultimate tensile strength, and procedural efficiency. The findings demonstrate that the Elsaftawy six-strand repair, constructed from widely available braided polyester in 4-0 diameter, showed no statistically significant differences in the measured biomechanical outcomes compared with the established Jin Bo Tang looped six-strand technique. Both the Elsaftawy and Jin Bo Tang repairs demonstrated significantly greater resistance to 2 mm gap formation and higher ultimate loads than the modified four-strand Kessler and FiberLoop^®^ repairs.

The ability of a repair to resist an early 2 mm gap is of particular clinical relevance. Even minimal gapping increases gliding resistance, promotes adhesion formation, and is strongly associated with poorer postoperative finger motion [3]. In this study, the Elsaftawy repair withstood a 2 mm gap force of 44.6 N, compared with 45.7 N for the Jin Bo Tang repair and 34.3 N for the Kessler repair. Both Elsaftawy and Jin Bo Tang techniques demonstrated significantly greater resistance to gap formation than the Kessler repair (*p* < 0.05), which aligns with previously published evidence demonstrating the mechanical superiority of higher-strand-count constructs [13,14]. FiberLoop^®^ demonstrated a lower value (29.4 N) and showed significantly lower resistance to gap formation compared with both Elsaftawy and Jin Bo Tang repairs, while not differing significantly from the Kessler repair.

The ultimate tensile strength results followed a similar pattern. The Elsaftawy repair demonstrated a mean ultimate load of 71.4 N, comparable to the Jin Bo Tang repair (71.8 N), with no significant difference between these techniques. Both six-strand repairs exhibited substantially greater ultimate tensile strength than the FiberLoop^®^ (52.0 N) and Kessler (42.9 N) repairs. Statistically significant differences were observed between each six-strand technique and both FiberLoop^®^ and Kessler repairs, whereas FiberLoop^®^ and Kessler did not differ significantly from each other. These findings support the concept that the biomechanical behavior of a tendon repair is influenced more by its structural architecture and load-distribution pattern than by suture caliber alone, provided the material has adequate baseline tensile capacity. This is consistent with prior studies highlighting the importance of transverse components, symmetric strand distribution, and multi-vector tensioning in optimizing load transfer across a core repair [14]. Similar findings were reported by Silva et al., who compared braided polyester and FiberWire^®^ sutures using the same modified Pennington repair in human flexor digitorum profundus tendons [15]. Despite differences in frictional properties, no significant differences were observed in gliding resistance or repair strength between the materials [15].

A notable finding of this study was the marked difference in failure mechanisms between groups. FiberLoop^®^ repairs failed predominantly via suture pullout, while all six-strand techniques demonstrated a rupture-dominant pattern, indicating more robust tendon–suture interaction. Pullout suggests limitations in core purchase or load-sharing geometry rather than insufficient material strength, a possibility supported by reports showing that UHMWPE constructs can perform poorly when the repair architecture does not permit effective load distribution [6,16]. By contrast, the symmetric architecture and pretensioning sequence of the Elsaftawy repair appear to promote more uniform force transmission across the repair site.

The Elsaftawy repair, by virtue of using affordable and universally available braided polyester rather than looped sutures, directly addresses the practical limitations associated with the Jin Bo Tang technique in many European and global settings. Loop-suture availability remains inconsistent, and costs can be prohibitive in cases involving multiple tendon injuries. FiberLoop^®^ offers reduced suturing time but requires a demanding 16-step sequence and has limited accessibility in lower-resource environments. In this context, the Elsaftawy repair offers a potential alternative using standard non-looped sutures, although its reproducibility across surgeons and clinical effectiveness remain to be established.

Despite the emphasis on biomechanics, the time required to complete each repair remains an important factor in busy surgical practices. FiberLoop^®^ was significantly faster than all other techniques, but this efficiency came at the cost of lower biomechanical robustness. No statistically significant differences in suturing time were observed between the Elsaftawy, Jin Bo Tang, and Kessler repairs under the tested laboratory conditions. However, this finding does not establish comparable operative efficiency in clinical practice, where restricted access within human zone II may affect the time required for each technique.

The gap resistance and ultimate load observed in this study support further investigation of the Elsaftawy repair for potential use with early active motion rehabilitation. However, the absence of cyclic fatigue testing and gliding resistance assessments is a substantial limitation when considering its safety for this purpose. Single load-to-failure testing cannot establish repair durability under repetitive loading or characterize friction during tendon excursion through the pulley system. Cyclic loading tests and gliding assessments, followed by clinical validation, are needed before these findings can support recommendations for early active motion rehabilitation.

This study has several limitations. Although porcine flexor tendons are a validated biomechanical model, they do not fully replicate the anatomy or gliding constraints of the human zone II environment. Moreover, they do not perfectly reproduce human collagen density, microstructure, or viscoelastic behavior. As such, absolute force values may not directly translate to clinical settings. Therefore, these results should be interpreted as comparative biomechanical findings under the tested laboratory conditions and should not be used alone to establish safe loading thresholds for postoperative rehabilitation in humans. All repairs were performed by a single, highly experienced hand surgeon under controlled laboratory conditions. Although this minimized inter-operator variability, it limits the generalizability of the findings to surgeons with different levels of experience. The learning curve and reproducibility of each technique across operators were not evaluated. Therefore, the observed repair strength and suturing times may not reflect those achieved by less experienced surgeons. Suturing times were measured in exposed ex vivo tendons and may underestimate those required within the confined anatomy of human zone II. The relative time differences between techniques may also change under these clinical constraints. The study did not include cyclic fatigue testing or gliding resistance assessments. The single load-to-failure protocol does not reproduce the repetitive loading and tendon excursion encountered during early motion rehabilitation. Consequently, the findings do not establish the ability of these repairs to maintain gap resistance and smooth tendon gliding during repeated movement. Additionally, only core repairs were evaluated, without epitendinous augmentation, to isolate the performance of the core constructs. This design may underestimate the strength and gap resistance of clinically augmented repairs. It also prevents assessment of how epitendinous augmentation affects repair bulk and gliding resistance. Specifically, we did not assess the bulk of the non-looped Elsaftawy six-strand repair beneath intact pulleys, either alone or with epitendinous augmentation. Whether the augmented construct would increase friction or impair tendon passage remains unknown and requires direct evaluation. Therefore, the observed differences between core repairs may not fully reflect those between complete repairs used in clinical practice. This study also lacks human cadaveric or clinical validation. Testing isolated porcine tendons did not allow assessment of tendon passage through intact pulleys, adhesion formation, or biological healing. These factors may affect postoperative tendon excursion and functional recovery, which cannot be predicted from initial repair strength alone. Lastly, the sample size of 15 tendons per group may have provided insufficient statistical power to detect small biomechanical differences between techniques. Therefore, the absence of statistically significant differences, particularly between the Elsaftawy and Jin Bo Tang repairs, should not be interpreted as evidence of biomechanical equivalence. Further studies are needed to assess gliding resistance, repair bulk beneath intact pulleys, learning curve characteristics, and in vivo tendon healing behavior. Ongoing cadaveric work and future clinical trials will be essential to validate these findings in human tissue and determine the translational impact on postoperative outcomes.

## 5. Conclusions

In conclusion, the Elsaftawy six-strand flexor tendon repair showed no statistically significant differences in the measured biomechanical outcomes compared with the Jin Bo Tang technique while using standard non-looped braided polyester sutures. The Elsaftawy repair demonstrated high mechanical performance, with an ultimate tensile strength of 71.4 N and resistance to 2 mm gap formation of 44.6 N. These values were comparable to those observed for the Jin Bo Tang repair, with no significant differences between the two techniques, and were higher than those recorded for traditional Kessler and FiberLoop^®^ repairs. Despite its enhanced strength, the Elsaftawy technique showed no significant differences in suturing time compared with Jin Bo Tang and Kessler repairs. Moreover, its symmetrical, pretensioned architecture promotes failure primarily by suture rupture rather than pullout. These findings support further evaluation of the Elsaftawy repair, but do not establish its reproducibility across surgeons or its suitability for early motion rehabilitation protocols. Future studies involving surgeons with different levels of experience, including general surgeons and trainees, are needed to assess the learning curve and reproducibility of the technique. Its use of accessible suture material makes it especially valuable in global surgery contexts, where material availability and procedural simplicity are critical. Further cadaveric and clinical studies will be needed to assess tendon passage through pulleys, adhesion formation, and long-term functional outcomes, but these results support its potential as a globally scalable solution for flexor tendon repair.

## Figures and Tables

**Figure 1:**
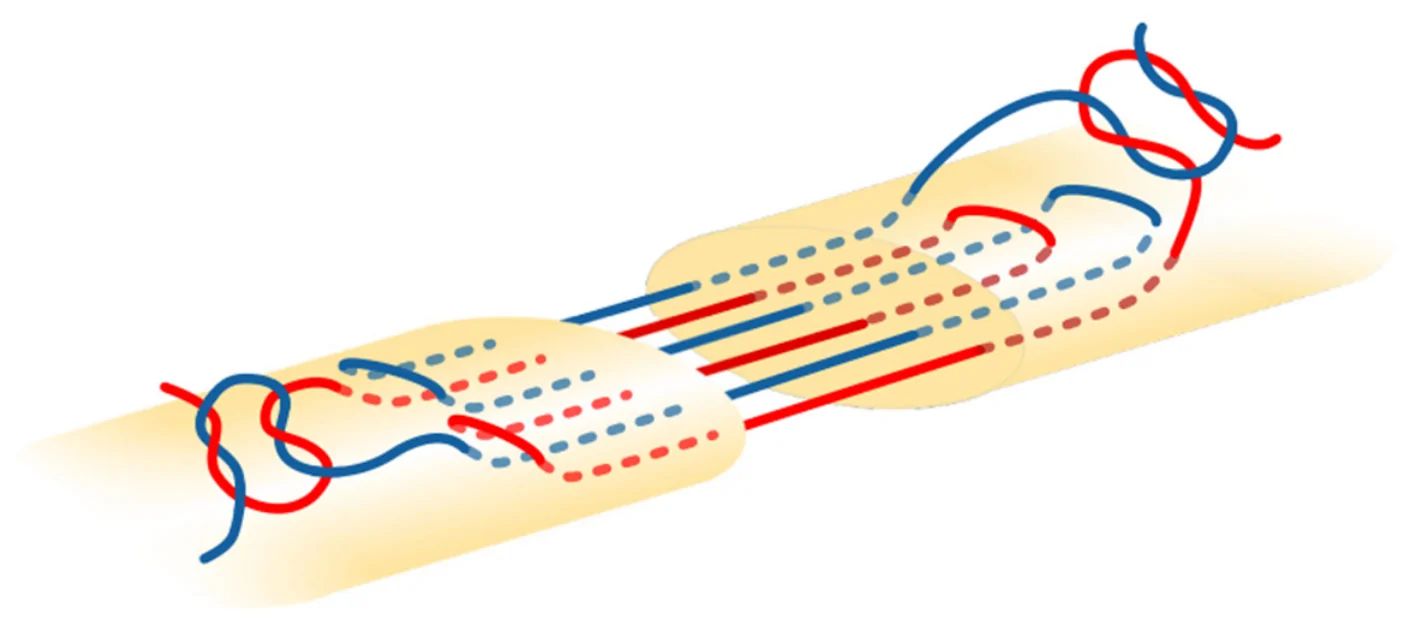
Scheme illustrating the Elsaftawy suture technique for tendon repair.

**Figure 2:**
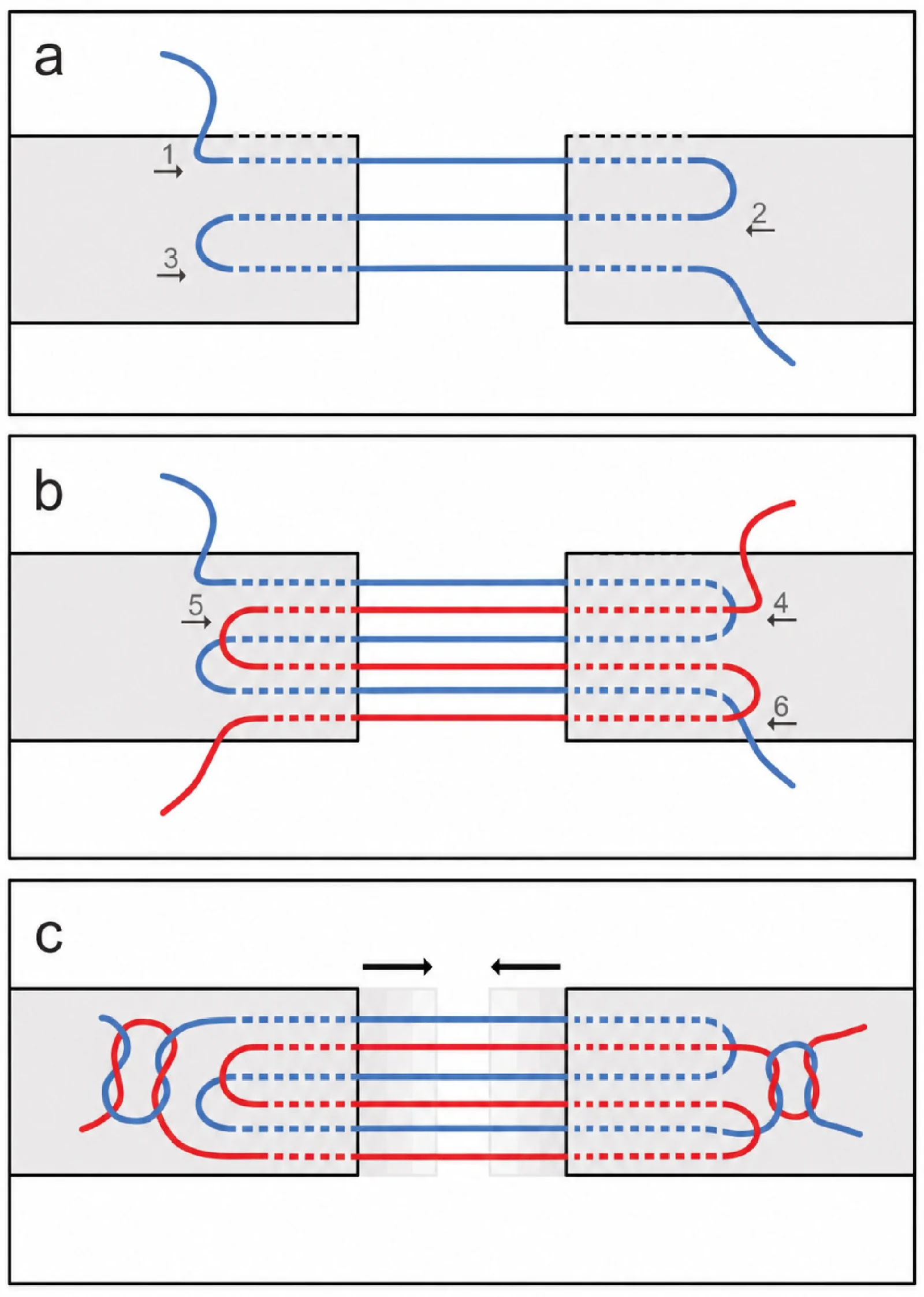
Scheme presenting a step-by-step guide of the Elsaftawy suture technique for tendon repair. (**a**) Placement of the first three suture strands. (**b**) Placement of three additional strands to complete the symmetric six-strand construct. (**c**) Tendon-ends approximation.

**Figure 3:**
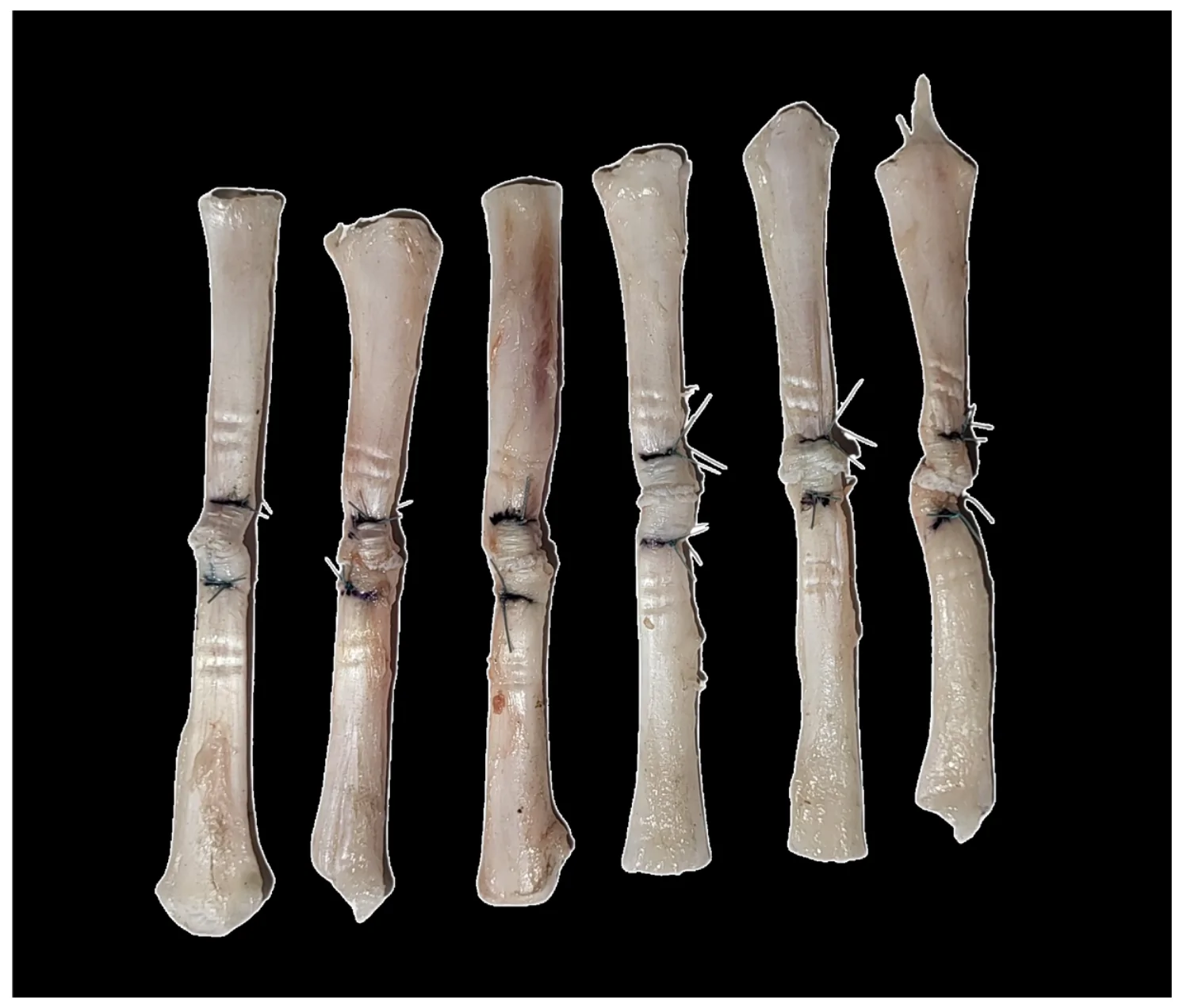
Photograph showing the sutured tendons prior to biomechanical testing.

**Figure 4:**
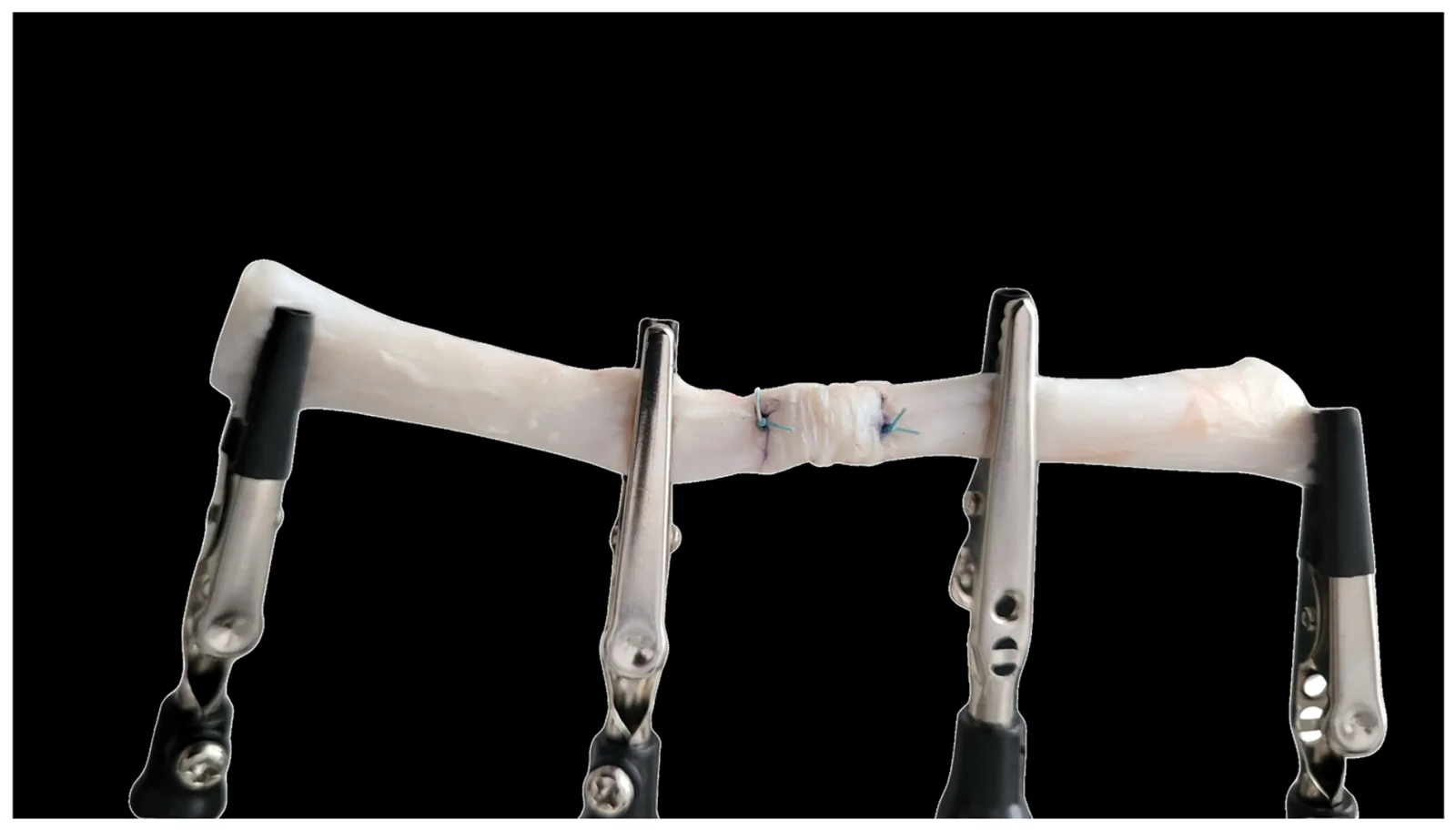
Photograph showing the sutured tendon prepared for biomechanical testing.

**Figure 5:**
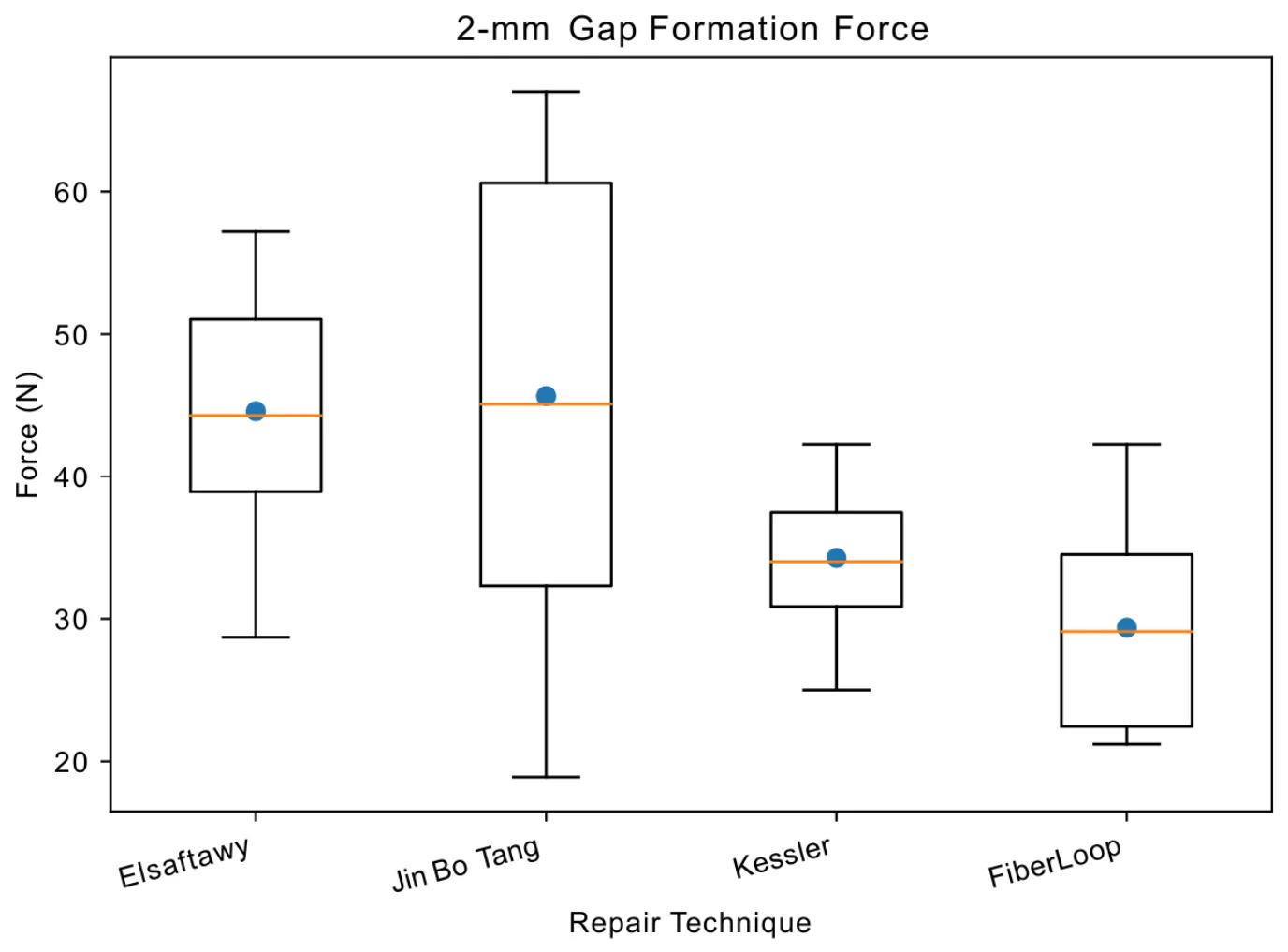
Boxplot showing group distributions in the 2 mm gap formation force analysis.

**Figure 6:**
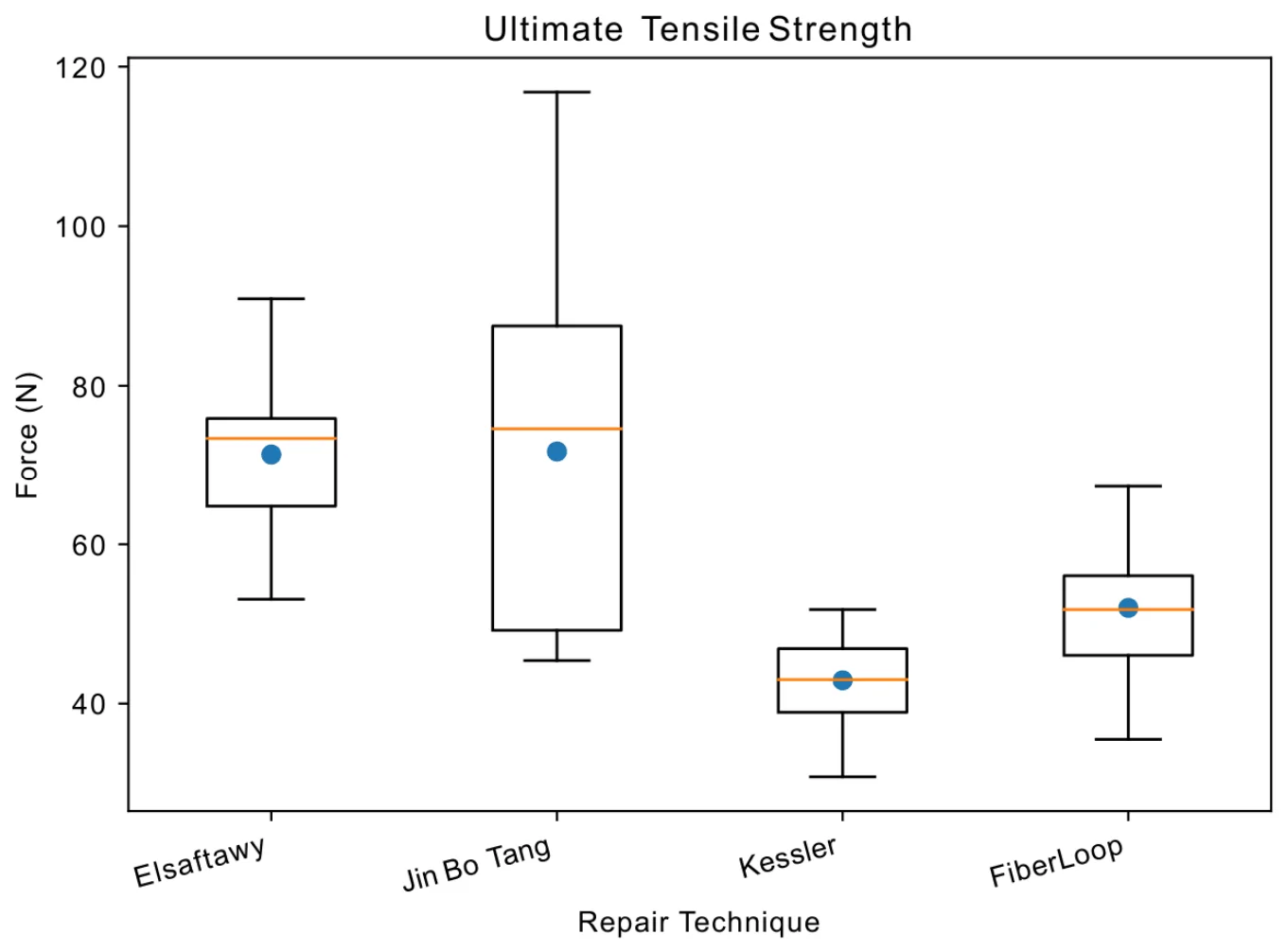
Boxplot showing group distributions in the ultimate tensile strength analysis.

**Figure 7:**
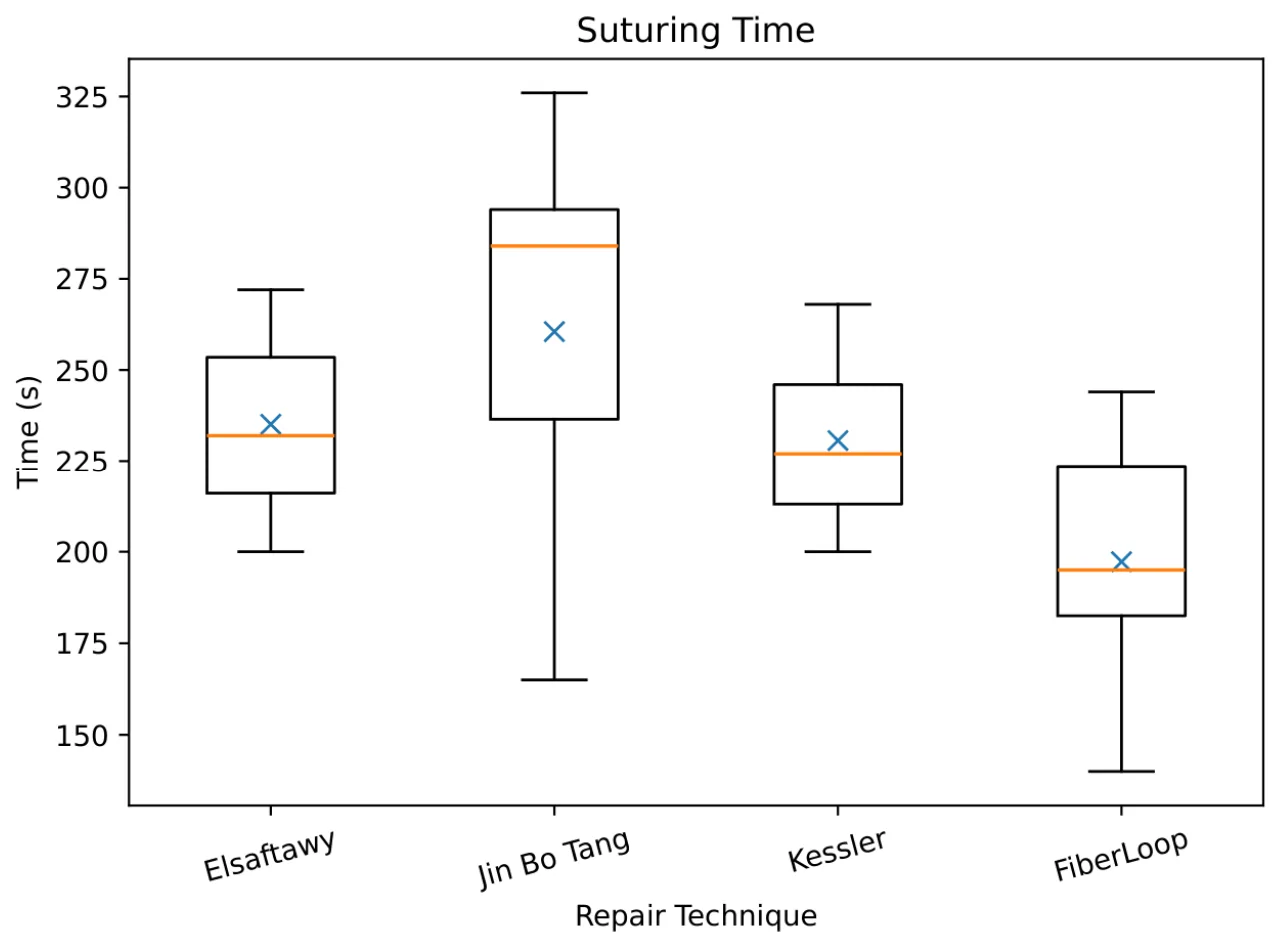
Boxplot showing group distributions in the suturing time analysis.

**Table 1:** The established means and standard deviations for each category across all groups.

Repair Technique	*N*	2-mm Gap [N]	Ultimate Load [N]	Suturing Time [s]
Elsaftawy 6-strand (4-0)	15	44.6 ± 7.9	71.4 ± 9.6	235.1 ± 23.3
Jin Bo Tang 6-strand (4-0 looped)	15	45.7 ± 16.7	71.8 ± 22.0	260.5 ± 48.1
Kessler 4-strand (4-0)	15	34.3 ± 5.9	42.9 ± 5.6	230.7 ± 20.7
FiberLoop^®^ UHMWPE (4-0 looped)	15	29.4 ± 7.1	52.0 ± 10.1	197.3 ± 29.8

**Table 2:** Tukey HSD pairwise comparisons between groups regarding the 2 mm gap formation force [N]. Comparisons with *p*-values < 0.05 are highlighted in bold.

Comparison	Mean Difference	*p*-Value	Interpretation
Elsaftawy vs. FiberLoop^®^	**−15.23**	**0.0009**	Elsaftawy significantly stronger
Elsaftawy vs. Jin Bo Tang	1.07	0.9920	No significant difference
Elsaftawy vs. Kessler	**−10.35**	**0.0397**	Elsaftawy significantly stronger
FiberLoop^®^ vs. Jin Bo Tang	**16.29**	**0.0004**	Jin Bo Tang significantly stronger
FiberLoop^®^ vs. Kessler	4.88	0.5711	No significant difference
Jin Bo Tang vs. Kessler	**−11.41**	**0.0191**	Jin Bo Tang significantly stronger

**Table 3:** Tukey HSD pairwise comparisons between groups regarding the ultimate tensile strength [N]. Comparisons with *p*-values < 0.05 are highlighted in bold.

Comparison	Mean Difference	*p*-Value	Interpretation
Elsaftawy vs. FiberLoop^®^	**−19.37**	**0.0011**	**Elsaftawy significantly stronger**
Elsaftawy vs. Jin Bo Tang	0.38	0.9998	No significant difference
Elsaftawy vs. Kessler	**−28.47**	**<0.0001**	**Elsaftawy significantly stronger**
FiberLoop^®^ vs. Jin Bo Tang	**19.75**	**0.0009**	**Jin Bo Tang significantly stronger**
FiberLoop^®^ vs. Kessler	−9.10	0.2517	No significant difference
Jin Bo Tang vs. Kessler	**−28.85**	**<0.0001**	**Jin Bo Tang significantly stronger**

**Table 4:** Tukey HSD pairwise comparisons between groups regarding the suturing time [s]. Comparisons with *p*-values < 0.05 are highlighted in bold.

Comparison	Mean Difference	*p*-Value	Interpretation
Elsaftawy vs. FiberLoop^®^	**−37.87**	**0.0115**	**FiberLoop significantly faster**
Elsaftawy vs. Jin Bo Tang	25.40	0.1491	No significant difference
Elsaftawy vs. Kessler	−4.47	0.9813	No significant difference
FiberLoop^®^ vs. Jin Bo Tang	**63.27**	**<0.0001**	**FiberLoop significantly faster**
FiberLoop^®^ vs. Kessler	**33.40**	**0.0316**	**FiberLoop significantly faster**
Jin Bo Tang vs. Kessler	−29.87	0.0657	No significant difference

**Table 5:** The results of the suture failure mechanisms across all groups.

Repair Technique	Total N	Pullout		Rupture	
		N	%	N	%
Elsaftawy 6-strand (4-0)	15	4	26.7%	11	73.3%
Jin Bo Tang 6-strand (4-0 looped)	15	4	26.7%	11	73.3%
Kessler 4-strand (4-0)	15	0	0.0%	15	100.0%
FiberLoop^®^ UHMWPE (4-0 looped)	15	11	73.3%	4	26.7%

## Data Availability

The data presented in this study are available upon request from the corresponding author.

## Supplementary Materials

The following supporting information can be downloaded at: https://www.mdpi.com/article/10.3390/jcm15197449/s1, Table S1: The ARRIVE guidelines 2.0: author checklist.
