# Supplementary material for: A New Accessible Six-Strand Flexor Tendon Repair Technique: A Four-Group Comparative Study on a Porcine Model
Source: J Clin Med. 2026 Sep 24;15(19):7449. doi: 10.3390/jcm15197449 (PMC13666442; doi:10.3390/jcm15197449)
Supplement: Supplementary file 1 [file jcm-15-07449-s001.zip › jcm-4489751-supplementary.pdf]

NOTE: Please save this file locally before filling in the table, DO NOT work on the file within your internet browser as changes will not be saved. Adobe Acrobat Reader (available free here) is recommended for completion.

The ARRIVE guidelines 2.0: author checklist

The ARRIVE Essential 10

These items are the basic minimum to include in a manuscript. Without this information, readers and reviewers cannot assess the reliability of the findings.

Item		Recommendation	Section/line number, or reason for not reporting
**Study design**	1	For each experiment, provide brief details of study design including:	
		a. The groups being compared, including control groups. If no control group has been used, the rationale should be stated.	Methods: Study Design.
		b. The experimental unit (e.g. a single animal, litter, or cage of animals).	Methods: Study Design. See note 1.
**Sample size**	2	a. Specify the exact number of experimental units allocated to each group, and the total number in each experiment. Also indicate the total number of animals used.	Results; Tables 1, 5. 15/group; 60 tendons. See note 2.
		b. Explain how the sample size was decided. Provide details of any *a priori* sample size calculation, if done.	Not reported. See note 2.
**Inclusion and exclusion criteria**	3	a. Describe any criteria used for including and excluding animals (or experimental units) during the experiment, and data points during the analysis. Specify if these criteria were established *a priori*. If no criteria were set, state this explicitly.	Methods: Specimen Preparation. See note 3.
		b. For each experimental group, report any animals, experimental units or data points not included in the analysis and explain why. If there were no exclusions, state so.	Results, opening paragraph: no specimen loss; all 60 tested.
		c. For each analysis, report the exact value of *n* in each experimental group.	Results; Tables 1, 5: n = 15 per group.
**Randomisation**	4	a. State whether randomisation was used to allocate experimental units to control and treatment groups. If done, provide the method used to generate the randomisation sequence.	Methods: Study Design; computer-generated randomisation sequence.
		b. Describe the strategy used to minimise potential confounders such as the order of treatments and measurements, or animal/cage location. If confounders were not controlled, state this explicitly.	Methods: Suturing Technique; Biomechanical Testing. See note 4.
**Blinding**	5	Describe who was aware of the group allocation at the different stages of the experiment (during the allocation, the conduct of the experiment, the outcome assessment, and the data analysis).	Not reported. See note 5.
**Outcome measures**	6	a. Clearly define all outcome measures assessed (e.g. cell death, molecular markers, or behavioural changes).	Methods: Suturing Technique; Biomechanical Testing.
		b. For hypothesis-testing studies, specify the primary outcome measure, i.e. the outcome measure that was used to determine the sample size.	Introduction; Methods: Biomechanical Testing. See note 6.
**Statistical methods**	7	a. Provide details of the statistical methods used for each analysis, including software used.	Methods: Statistical Analysis.
		b. Describe any methods used to assess whether the data met the assumptions of the statistical approach, and what was done if the assumptions were not met.	Methods: Statistical Analysis; Results, opening paragraph.
**Experimental animals**	8	a. Provide species-appropriate details of the animals used, including species, strain and substrain, sex, age or developmental stage, and, if relevant, weight.	Methods: Specimen Preparation. See note 8.
		b. Provide further relevant information on the provenance of animals, health/immune status, genetic modification status, genotype, and any previous procedures.	Methods: Specimen Preparation. See note 8.
**Experimental procedures**	9	For each experimental group, including controls, describe the procedures in enough detail to allow others to replicate them, including:	Methods: all subsections; Figures 1-4.
		a. What was done, how it was done and what was used.	Methods: Specimen Preparation; Biomechanical Testing.
		b. When and how often.	Methods: Specimen Preparation. See note 9.
		c. Where (including detail of any acclimatisation periods).	
		d. Why (provide rationale for procedures).	Introduction; Methods: Specimen Preparation; Suturing Technique.
**Results**	10	For each experiment conducted, including independent replications, report:	Results; Tables 1, 5; Figures 5-7.
		a. Summary/descriptive statistics for each experimental group, with a measure of variability where applicable (e.g. mean and SD, or median and range).	
		b. If applicable, the effect size with a confidence interval.	Tables 2-4: mean differences. CIs not reported. See note 10.

The Recommended Set

These items complement the Essential 10 and add important context to the study. Reporting the items in both sets represents best practice.

Item		Recommendation	Section/line number, or reason for not reporting
**Abstract**	11	Provide an accurate summary of the research objectives, animal species, strain and sex, key methods, principal findings, and study conclusions.	Abstract.<br>See note 11.
**Background**	12	a. Include sufficient scientific background to understand the rationale and context for the study, and explain the experimental approach.	Introduction.
		b. Explain how the animal species and model used address the scientific objectives and, where appropriate, the relevance to human biology.	Methods: Specimen Preparation;<br>Discussion.
**Objectives**	13	Clearly describe the research question, research objectives and, where appropriate, specific hypotheses being tested.	Introduction, final paragraph.
**Ethical statement**	14	Provide the name of the ethical review committee or equivalent that has approved the use of animals in this study, and any relevant licence or protocol numbers (if applicable). If ethical approval was not sought or granted, provide a justification.	Methods: Specimen Preparation;<br>Institutional Review Board Statement.<br>No live animals used.
**Housing and husbandry**	15	Provide details of housing and husbandry conditions, including any environmental enrichment.	Not applicable:<br>ex vivo slaughterhouse tissue;<br>no live animals used.
**Animal care and monitoring**	16	a. Describe any interventions or steps taken in the experimental protocols to reduce pain, suffering and distress.	Not applicable:<br>no live animals used.
		b. Report any expected or unexpected adverse events.	Not applicable:<br>no live animals used.
		c. Describe the humane endpoints established for the study, the signs that were monitored and the frequency of monitoring. If the study did not have humane endpoints, state this.	Not applicable:<br>no live animals used.
**Interpretation/ scientific implications**	17	a. Interpret the results, taking into account the study objectives and hypotheses, current theory and other relevant studies in the literature.	Discussion.
		b. Comment on the study limitations including potential sources of bias, limitations of the animal model, and imprecision associated with the results.	Discussion: paragraph starting<br>"This study has several limitations."
**Generalisability/ translation**	18	Comment on whether, and how, the findings of this study are likely to generalise to other species or experimental conditions, including any relevance to human biology (where appropriate).	Discussion: limitations and concluding paragraph.
**Protocol registration**	19	Provide a statement indicating whether a protocol (including the research question, key design features, and analysis plan) was prepared before the study, and if and where this protocol was registered.	Not reported.<br>See note 19.<br>Methods, Specimen Preparation
**Data access**	20	Provide a statement describing if and where study data are available.	Data Availability Statement:<br>available on request from the corresponding author.
**Declaration of interests**	21	a. Declare any potential conflicts of interest, including financial and non-financial. If none exist, this should be stated.	Conflicts of Interest:<br>none declared.
		b. List all funding sources (including grant identifier) and the role of the funder(s) in the design, analysis and reporting of the study.	Funding:<br>no financial support received.

Checklist completion notes

Manuscript jcm-4489751 | Revised version supplied by the authors

Section references follow the supplied manuscript headings. These notes form part of the completed checklist. "Not reported" identifies information absent from the supplied text; it does not imply that a procedure was not performed.

1b - Experimental unit

The experimental unit is an individual tendon. The number of source animals and whether multiple tendons came from the same animal are not reported; independence at the animal level cannot be assessed.

2a-2b - Sample size

Tables 1 and 5 report 15 tendons per group (60 total). The number of source animals is not reported. The manuscript provides neither a sample-size rationale nor an a priori calculation. The Discussion acknowledges limited power to detect small differences.

3a - Eligibility criteria

Specimen Preparation describes exclusion of tendons with visible degeneration, fraying, or structural inconsistency. It does not explicitly state whether these criteria were established a priori or specify criteria for excluding individual analytical data points.

4b - Potential confounders

A single surgeon, standardized repair parameters, tissue handling and mechanical testing are described. The order of repairs and measurements, and any strategy to control order-related confounding, are not reported.

5 - Blinding

Awareness of group allocation during allocation, experimental procedures, outcome assessment and data analysis is not reported.

6b - Primary outcome for sample-size determination

The Introduction lists the outcomes, and Biomechanical Testing defines the mechanical endpoints. No outcome is identified as the basis for a sample-size calculation.

8a-8b - Source animal characteristics

Specimen Preparation identifies skeletally mature domestic pigs (Sus scrofa domestica), routine slaughter and a licensed abattoir. Breed/strain, sex, exact age, weight, health/immune status, genetic status and previous procedures are not reported. No live animals were used or sacrificed specifically for this study.

9c - Experimental location

A licensed abattoir is mentioned for tissue collection, but its identity/location and the site of repair and biomechanical testing are not explicitly stated. Author affiliations alone do not establish the experimental location. Acclimatisation is not applicable to ex vivo tissue.

10b - Effect estimates

Tables 2-4 provide pairwise mean differences and p-values, but no confidence intervals. This item is therefore only partially reported.

11 - Abstract

The Abstract reports the porcine model, objectives, methods, main findings and conclusions. Source-animal sex and breed/strain are not specified.

19 - Protocol registration

The manuscript does not state whether a protocol was prepared before the study or whether it was registered. Absence of reporting is not evidence that no protocol or registration existed.
